# Antidromic vasodilatation and the migraine mechanism

**DOI:** 10.1007/s10194-011-0408-3

**Published:** 2011-12-27

**Authors:** Pierangelo Geppetti, Eleonora Rossi, Alberto Chiarugi, Silvia Benemei

**Affiliations:** 1 Headache Centre, Careggi University Hospital, University of Florence, Florence, Italy; 2 Department of Preclinical and Clinical Pharmacology, University of Florence, Viale Pieraccini 6, 50139 Florence, Italy

**Keywords:** Antidromic vasodilatation, Neurogenic inflammation, CGRP, Migraine, Chronic migraine

## Abstract

Despite the fact that an unprecedented series of new discoveries in neurochemistry, neuroimaging, genetics and clinical pharmacology accumulated over the last 20 years has significantly increased our current knowledge, the underlying mechanism of the migraine headache remains elusive. The present review article addresses, from early evidence that emerged at the end of the nineteenth century, the role of ‘antidromic vasodilatation’ as part of the more general phenomenon, currently defined as neurogenic inflammation, in the unique type of pain reported by patients suffering from migraine headaches. The present paper describes distinctive orthodromic and antidromic properties of a subset of somatosensory neurons, the vascular- and neurobiology of peptides contained in these neurons, and the clinical–pharmacological data obtained in recent investigations using provocation tests in experimental animals and human beings. Altogether, previous and recent data underscore that antidromic vasodilatation, originating from the activation of peptidergic somatosensory neurons, cannot yet be discarded as a major contributing mechanism of the throbbing head pain and hyperalgesia of migraine.

## Antidromic vasodilatation, axon reflex and hyperalgesia

### Early studies

Pioneering physiological studies at the end of the nineteenth century through the beginning of the twentieth postulated the existence of a double function of a subset of sensory, afferent neurons with cell bodies located in the dorsal root ganglia (DRG). We owe Bayliss [1] for the experiments, performed principally on the dog, and his brilliant interpretation that led him to conclude:

1. ‘There are nerve-fibres in the posterior roots of the 5th, 6th, and 7th lumbar and 1st sacral nerves, excitation of which, when cut away from the spinal cord, gives rise to vascular dilatation in the hind-limb of the same side. The excitation may be either electrical, mechanical, chemical, or thermal, and of these, mechanical excitation is most effective.’
2. ‘They do not degenerate when cut between spinal cord and posterior root ganglion, hence they are not spinal efferent fibres. They do degenerate when posterior root ganglia are extirpated, hence their trophic centres are in these ganglia.’
3. ‘They are, in fact, identical with the ordinary sensory afferent posterior root-fibres; the name “antidromic” is suggested for the process by which nerve-fibres convey impulses in a direction contrary to that assumed by the Bell–Majendie law, when such impulses produce effects in the organs at the origin of such fibres, e.g. when afferent fibres excited at their ends in the central nervous system produce vascular dilatation at their peripheral ends in the tissues of the body.’

### Antidromic vasodilatation

The biochemical substance(s) that mediates the flare response to injury or to capsaicin applied to the human skin has remained unidentified for more than a century. The discovery that a subset of primary sensory neurons express, and, from their peripheral terminals, release vasoactive neuropeptides has pointed to these substances as the mediators of the flare evoked by the activation of axon reflexes [2]. The tachykinin substance P (SP) and neurokinin A (NKA) and the longer 37 aminoacid peptide, calcitonin gene-related peptide (CGRP), are all found in a subset of neurons of the DRG, vagal and trigeminal (TG) ganglia. During the last 20 years cloning of their receptors and identification of selective antagonists [3–6] have provided powerful tools to unravel their functions when liberated from central and peripheral endings of pseudounipolar somatosensory neurons. The role of both tachykinins and CGRP released in the dorsal spinal cord at the level of the lamina I and II remains uncertain, as they do not seem to give a major contribution to nociceptive information or hyperalgesia. In contrast, in rodents compelling evidence indicates that SP/NKA mediate the plasma protein extravasation, and CGRP is responsible for most of the vasodilatatory component of the inflammatory response evoked by stimulation of sensory nerve endings in peripheral tissues [2]. The observation that the CGRP receptor antagonist, telcagepant [7], markedly reduced the increase in forearm skin blood flow evoked by topical application of capsaicin [8] points to CGRP as the major, if not the sole, mediator of the neurogenic dilator response resulting from sensory nerve terminal activation in humans.

### Hyperalgesia and flare

About 40 years after the seminal observation of William Bayliss, Lewis [9] addressed the issue of how the skin surrounding an injured site becomes painful, by hypothesizing the existence of “nocifensor” nerve fibres. According to Lewis’ hypothesis, these ‘specialized’ fibres would detect information about a localized injury and spread this information both to surrounding sensory afferents that encoded pain, thereby causing hyperalgesia, and to blood vessels to cause local dilatation leading to the flare response (reddening of the skin). With the term “axon reflex” Lewis [9] depicted the hypothesis that from one portion of a widely branching sensory fibre which responded to the injury, action potentials propagated antidromically to collateral nerve fibres where they liberated a chemical substance, which in turn caused the flare and enhanced sensitivity of other sensory axons responsible for pain. This proposal originally generated by the study of injurious stimuli [9] was substantially confirmed by other authors [10–12], who used capsaicin as the stimulus which promotes vasodilatation and hyperalgesia.

The hypothesis [13–15] that inputs from C nociceptors lead to presynaptic depolarization, strong enough to initiate action potentials, which antidromically propagate to the peripheral receptive fields of these fibres, is substantially rejected by the observation that blocking central nerve trunks [10] or cutting nerves [1, 9] does not block the flare. The block of the proximal nerve with local anaesthetic did not prevent the spread of flare around the injection site, but did prevent the development of hyperalgesia some hours later [10]. Thus, central nervous system neurons encoding painful inputs must receive some decisive input from the nerve fibres of the hyperalgesic region during the early phases of exposure to the stimulus. The proposed model [10] was that chemospecific peripheral nerve fibres either widely branching or functionally coupled together in the periphery may sensitize low- and high-threshold mechanoreceptive interneurons in the dorsal horn. A subcategory of mechanically sensitive C-fibre neurons may directly contribute and also facilitate Aδ-fibre neurons to produce hyperalgesia [16].

It should be underlined that subsequent studies which used thermography [11] or laser Doppler flowmetry [12] to determine the area of flare/vasodilatation after the injection of capsaicin into the human skin showed a much larger area than that previously identified by the sole visual inspection of the area of reddening. A strict correspondence between the areas of flare, and mechanical and heat hyperalgesia induced by intradermal capsaicin injection, suggested that all three phenomena are the consequence of neural factors that operate peripherally [11, 12]. Nevertheless, whether an intimate functional link exists between areas of flare and mechanical and heat hyperalgesia is still controversial. In addition, the identity of the chemical mediator(s) that directly or indirectly establishes, or contributes to, hyperalgesia in the area of the axon flare, and particularly if this may be considered CGRP or not, remains to be determined. For example, in rats, if CGRP injection in the dorsal skin was not found to cause hyperalgesia [17], repeated intraplantar injections of low doses of CGRP have been shown to induce hyperalgesia [18].

### Allodynia, hyperalgesia and migraine

On the basis of the progress of allodynia in a migraine patient, it was proposed [19] that during a migraine attack, soon after the activation of peripheral nociceptors, these same neurons become sensitized, and the barrage of impulses deriving from peripheral input recruits second and third order neurons, then spreading to the contralateral side of the head and to ipsilateral anatomical sites at a distance from the head. Experimental evidence and related models of sensitization, exposed in the previous paragraph, can be applied to migraine, postulating that the sensitization process, which eventually results in allodynia and hyperalgesia, initiates at peripheral sites. Although not conclusively demonstrated, but suggested by positron emission tomography neuroimaging studies [20], the onset of a migraine attack might take place in and extracranial deep brain structures. Although in experimental animals there is no evidence for mechanical activation of nociceptive afferents during vessels dilatation, novel clinical findings suggest that the headache phase depends on nociceptive input from perivascular sensory nerve terminals, as shown by: (1) the temporal association of pain with dilatation of intra- and extracranial arteries; and (2) the temporal association of pain resolution by antimigraine drugs with constriction of dilated extracranial arteries [21]. This, together with a series of headache/migraine provocation tests in healthy volunteers and migraine patients, has led to the conclusion that migraine can be explained to patients as ‘a disorder of the brain, and that the headache originates in the sensory fibres that convey pain signals from intracranial and extracranial blood vessels’ [22].

## Features and mechanisms of neurogenic inflammation

### Tachykinins

The introduction of experimental neurobiology and pharmacology of capsaicin, with its unique features to activate and desensitize a specific subset of C- and Aδ-fibre nociceptors, has been instrumental for the identification of the key contribution of these neurons to neurogenic inflammation [2]. The major vascular action of CGRP action (conserved throughout the mammal species, including man) is the vasodilatation of large and small size arteries via direct activation of the calcitonin receptor-like receptor (CLR) associated to the receptor activity-modifying protein-1 (RAMP1) [4], which are primarily located on the plasma membrane of arterial smooth muscle cells. Other functions of CGRP, however, irrelevant for the present discussion, are directed to relax smooth muscle cells in certain tissues, including those of the urinary tract or the intestine [2]. Species-specific actions, some of which are of proinflammatory nature, are mediated by SP and NKA through the activation of their receptors, namely the NK1, NK2 and NK3 receptors. These include plasma protein extravasation evoked by activation of NK1 receptors on endothelial cells of postcapillary venules, contraction of iris or bronchial smooth muscle, and secretion from seromucous glands. SP has also been proposed to accumulate neutrophils [23], activate macrophages [24], and stimulate mast cells. However, it should be underlined that there is no conclusive proof that endogenously released tachykinins (e.g. after capsaicin administration) can, through a receptor mediated mechanism, act on these pro-inflammatory cells. Rather, there is evidence that some of these actions are either indirect or mediated by a receptor-independent action [25].

Irrespective of the undisputable role of SP in rodent neurogenic plasma protein extravasation, the failure of NK1 receptor antagonists in the acute migraine attack convincingly discarded the hypothesis that meningeal plasma protein extravasation could be the underlying mechanism of migraine [26]. The ability to detect the capsaicin-evoked release of CGRP and the failure to measure that of SP from slices of human iris in vitro [27] and the observation that capsaicin injection into the human skin causes flare (mediated by CGRP) but not wheal (mediated by SP) [10] suggest that in humans SP is not released from peripheral endings of somatosensory neurons, or that it is released in amounts not sufficient to provoke the effects that it evokes in rodents.

### Calcitonin gene-related peptide

Thus, after excluding the contribution of sensory tachykinins, which in humans do not seem to exert any major role, the residual component of the neurogenic inflammatory response that might be implicated in the pathophysiology of the migraine attack is confined to CGRP and its vasomotor effects. The seminal observation that CGRP is increased in blood deriving from the cranial circulation during migraine attacks [28] and more importantly the ability of three chemically unrelated CGRP receptor antagonists to ameliorate the pain and the associated symptoms of the migraine attack [29–31] supports the view that CGRP-dependent effects are of paramount importance in the mechanism of migraine. In the context of the present article, the hypothesis that antidromic release of CGRP from peripheral terminals of trigeminal somatosensory neurons and its ensuing vascular effects play a role in migraine will be addressed.

## Molecular targets of migraine triggers

### TRPV1 stimulating agents

The importance of the vasodilator component of neurogenic inflammation in migraine is further supported by the observation that known triggers of migraine attacks produce neurogenic vasodilatation in experimental animal models. A large and variable number of stimuli may precipitate migraine [32]. Among them, stress with the ensuing post-stress phase is often described by patients as responsible for precipitating their headaches. Placing mice in a stressful condition was found to increase dural vascular permeability in a manner dependent on NK1 receptor, thus indicating the involvement of neurogenic inflammation [33]. In this model, CGRP-mediated responses were not measured, but due to the fact that upon stimulation of capsaicin-sensitive neurons both SP and CGRP are invariably co-released, it is possible that exposure to stress also results in the release of CGRP and CGRP-dependent vasodilatation.

Perfumes/odours, alcoholic beverages and cigarette smoke are also reported by about 30–40% of the patients as effective triggers of migraine attacks [32]. A few years ago ethanol was identified as a stimulus of the transient receptor potential vanilloid 1 (TRPV1) channel [34]. TRPV1, also known as the capsaicin receptor, belongs to a larger family of non-selective cation channels responsible for a series of pleiotropic biological responses [35]. TRPV1 is co-expressed with the neuropeptides, SP and CGRP in somatosensory neurons [36], and therefore its stimulation by capsaicin and other stimuli results in neuropeptide release and neurogenic inflammatory responses [2]. Ethanol action on TRPV1 appears to be indirect, as ethanol reduces the threshold temperature for TRPV1 activation (normally 42–43°C) by 8°C to 34–35°C. Therefore, in the presence of ethanol, the normal body temperature of 37°C is sufficient to activate the channel and to promote neurogenic inflammatory responses [34]. This unique property of ethanol may explain the burning quality (similar to that evoked by capsaicin) of the pain provoked by alcoholic beverages or tinctures when applied to wounded cutaneous surfaces or to oral and other types of mucosa. In meningeal arterial vessels of the guinea pig, the administration of an amount of alcohol corresponding to that contained in 3–4 glasses of wine produced a TRPV1-mediated and CGRP-dependent vasodilatation that has been suggested to explain the ability of alcoholic beverages to trigger migraine in susceptible individuals [37].

### TRPA1 stimulating agents

A substantial proportion of neurons that express TRPV1 also express the transient receptor potential ankyrin 1 (TRPA1) channel [38]. Among TRP channels, TRPA1 shows the unique feature to be activated (and accordingly, it has been proposed as a sensor of) by by-products of oxidative and nitrative stress [39]. Thus, exogenous agents such as formaldehyde, acrolein or crotonaldehyde (contained in cigarette smoke or environmental pollution) [40, 41], or endogenous compounds generated by the peroxidation of plasma membrane phospholipids, such as 4-hydroxynonenal and 4-oxononenal [42] are powerful stimulants of TRPA1. *Umbellularia californica* Nutt. is a tree indigenous to southwestern Oregon and Northern California. As known by native Americans, and first reported more than 100 years ago [43], exposure to *U. californica* (commonly known as the ‘headache tree’) can trigger violent headache crises [44].We recently described a case of cluster headache-like attacks triggered by the inhalation of the scent of *U. californica* [45]. However, the xenobiotic(s) contained in the plant scent, responsible for triggering headaches in migraineurs and in cluster headache patients, is unknown.

**Fig. 1:**
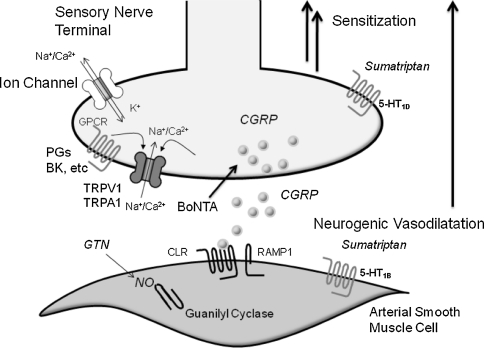
Neurogenic vasodilation in migraine. Activation of peptidergic trigeminal sensory neurons results in the release of calcitonin gene-related peptide (CGRP), which acting at the CGRP-like receptor (CLR) coupled to receptor activity-modifying protein-1 (RAMP1), relaxes smooth muscle cells of intra- and extracranial arteries. Exogenous or endogenous agonists of transient receptor potential vanilloid 1 (TRPV1) or ankyrin 1 (TRPA1) promote the release of CGRP. Prostaglandins (PGs), bradykinin (BK) or other proalgesic agents, either directly via activation of their specific G protein coupled receptors (GPCR) or indirectly (through channel activation) also contribute to neuropeptide release. Nitric oxide (NO) generated from glyceryl trinitrate (GTN) acts downstream to CGRP to evoke vasodilatation and probably headache. Neurogenic vasodilatation in addition to cause the headache may also contribute to neuronal sensitization. Sumatriptan and other triptans inhibit CGRP release acting at prejunctional serotonin 5-HT1D receptors or evoke arterial vasoconstriction via stimulation of 5-HT1B receptors on vascular smooth muscle. Botulinum neurotoxin type A (BoNTA) has also been shown to inhibit CGRP release

*U. californica* contains, as a major volatile constituent, umbellulone, a monoterpene ketone, with a strong, camphor-like odour, which exerts irritant effects in laboratory animals [44]. Umbellulone has recently been identified as an agonist of either the heterologously expressed human TRPA1 or native rat and mouse TG neurons TRPA1 [46]. It has also been reported that umbellulone application to the rat nasal mucosa causes, through TRPA1 stimulation, a CGRP-dependent meningeal vasodilatation [46]. These findings, and the observation that intranasal application of acrolein or of the TRPA1 agonist, allyl isothiocyanate, evokes a similar, CGRP-mediated meningeal vasodilatation [47], support the hypothesis that ammonium chloride, chlorine, cigarette smoke, and formaldehyde, all known triggers of migraine or cluster headache [32, 48–51], precipitate pain attacks by their recently identified ability to target neuronal TRPA1 and thereby releasing CGRP [39, 40, 52, 53]. Thus, emerging evidence supports the hypothesis that some triggers of migraine, and possibly cluster headache, act via a general mechanism which, by stimulating perivascular sensory nerve endings, releases the vasodilator peptide CGRP. All these various and chemically unrelated compounds stimulate TRPA1 or TRPV1 on cranial perivascular nerve fibres to activate a final common pathway, which is identical to that previously proposed as occurring in the skin following tissue injury [1, 9] or exposure to capsaicin [10, 11], and which recognizes antidromic release of CGRP and its vasodilatation/flare effect as the final step (Fig. 1).

## Neurogenic vasodilatation and migraine

### Vasodilatation

The seminal proposal by Graham and Wolff [54] who associated the headache phase of the migraine attack with the throbbing dilatation of the superficial temporal artery, and pain termination by ergotamine with a dramatic reduction in arterial pulsatility, has been subsequently challenged by the failure to measure any significant vasodilatation during migraine attacks either by using a laser Doppler flowmetry technique [55] or more recently in a magnetic resonance angiography (MRA) study [56]. However, more recent findings, obtained with a novel high-resolution direct MRA imaging technique [21] during headache and migraine attacks provoked by a 20 min CGRP infusion, suggest the reconsideration of the rejection of vasodilatation as a major contributing mechanism of migraine pain, and the resulting dismissal of Wolff’s hypothesis. In migraine patients, infusion of CGRP-evoked delayed, unilateral migraine pain associated with dilatation of the middle meningeal artery (MMA, with a more pronounced dilatation) and of the middle cerebral artery (MCA, with a less pronounced dilatation) on the pain side, but not on the pain-free side. In patients with bilateral migraine pain, bilateral dilatation of both the MMA and MCA was recorded [21]. Thus, the migraine pain was associated with arterial vasodilatation both with respect to the time course and the side of the event.

The series of enlightening clinical provocation tests in healthy volunteers and migraine patients by the Copenhagen group has greatly contributed to our current understanding of the role of CGRP and vasodilatation in migraine. CGRP infusion caused headaches and migraine-like attacks in migraineurs [57], although this effect, as those of other vasodilators, occurs when presumably the concentration of the vasodilator substance in blood is low or negligible. Further studies showed that in healthy volunteers, the CGRP receptor antagonist, olcegepant, significantly reduced the immediate and delayed headache evoked by CGRP administration [58]. If the ability of CGRP receptor activation or inhibition to provoke or abort, respectively, headache and migraine attacks has been well established, the site of action of CGRP to evoke head pain is unclear. It is possible that the absence of or the very limited [59] dilation of MCA evoked by CGRP infusion is due to the inability/poor ability of exogenously administered CGRP to cross the blood–brain barrier (BBB), and therefore to act in the intracerebral vascular compartment [60]. This conclusion is further supported by the observation that olcegepant (a CGRP antagonist of peptoid nature, which does not easily cross the BBB) [61, 62] had no effect on the slight increase in MCA vasodilatation, but inhibited the much larger dilation of extracerebral arteries, such as the superficial temporal and the radial arteries [58]. A mechanism similar to that found in patients with migraine without aura (MO) should occur in patients with migraine with aura (MA), as CGRP infusion triggered migraine-like attacks (some with aura) in patients suffering exclusively from MA [63]. However, CGRP infusion failed to induce migraine attack in patients affected by the familial hemiplegic migraine (FHM) with known mutations in the calcium channel, voltage-dependent, P/Q type, alpha 1A subunit, the ATPase, Na^+^/K^+^ transporting, alpha 2 (+) polypeptide genes or without known mutations [63]. Although the obviously small number of patients with FHM limits the robustness of the conclusion, these data suggest differences in the mechanism of head pain between patients with FHM and patients with MO/MA.

An emerging role for CGRP and neurogenic vasodilatation in chronic migraine is suggested by findings obtained by the injection of botulinum neurotoxin type A (BoNTA) into the rat craniofacial muscles, a procedure that decreased mechanical sensitization mediated by glutamate and CGRP-induced neurogenic vasodilatation [64]. Similar observation was previously obtained in the human skin where pretreatment with BoNTA reduced the pain and the neurogenic vasodilatation evoked by topical capsaicin application [65]. Thus, preclinical and clinical evidence suggests that BoNTA, recently introduced for the treatment of chronic migraine and medication overuse headache [66–68], may exert its beneficial effect by decreasing mechanical sensitivity of cranial muscle nociceptors through inhibition of glutamate release and by attenuating the release of CGRP from muscle nociceptors.

### Vasoconstriction

The antimigraine effect of sumatriptan has originally been linked to its ability to produce a rather selective contraction of cranial vessels as compared to other arteries in the heart and other tissues [69]. The hypothesis that meningeal neurogenic plasma protein extravasation could be responsible for the migraine attack [70], was strengthened by the ability of triptans to inhibit this type of inflammatory response in rodents, probably by reducing the release of SP/NKA [71]. Triptans have also been shown to inhibit the release of CGRP [71–73]. These mechanisms cannot be dismissed when considering the antimigraine effect of triptans. However, if the action of triptans was limited to the presynaptic inhibition of sensory neuropeptides, they should act as inhibitors of neurogenic vasodilatation or, in other terms, as indirect vasoconstrictors. As a consequence, triptans should not reduce the vasodilatation evoked by CGRP which, via the intracellular increase in cyclic adenylyl monophosphate, directly relaxes the arterial smooth muscle. In CGRP-evoked attacks, amelioration of the delayed migraine by sumatriptan administration was associated with the contraction of the dilated MMA, but not of the dilated MCA [21]. Thus, resolution of the migraine attack by sumatriptan was linked to the selective vasoconstriction of the extracranial MMA [21]. The proposal that exogenous CGRP does not dilate the MCA and that olcegepant does not inhibit the vasodilatation of the artery because neither CGRP nor olcegepant can easily cross the BBB [60] could be extended to sumatriptan. Its failure or poor capacity to constrict both normal and dilated MCA [21, 60] could be due to its poor ability to cross the BBB [74].

During a migraine attack occurring from several minutes to hours after the end of CGRP infusion, it is possible that exogenous CGRP (likewise many other vasodilator substances) targets a hitherto undefined central site that eventually results in sensory nerve activation and the release of endogenous CGRP. Under these circumstances, sumatriptan, acting on presynaptic 5-HT1D receptors might have inhibited a delayed CGRP release. However, this conclusion is challenged by three additional observations. Firstly, sumatriptan caused constriction of the MMA, non-dilated because it is either under baseline conditions [60] or on the pain-free side during a monolateral migraine attack [21]. Under these two conditions presumably there is no release of CGRP from perivascular sensory nerve terminals. Secondly, in healthy volunteers sumatriptan was found to reduce the early increase in MMA circumference, a response that, in contrast with the vasodilatation associated with the migraine-like attack observed in migraine patients, is closely associated with CGRP administration [60]. Thirdly, the high affinity CGRP receptor antagonist, olcegepant, which effectively reduced spontaneous [31] and CGRP-provoked [58] attacks, did not ameliorate migraine attacks evoked by the direct vasodilator, nitric oxide (NO) donor, and glyceryl trinitrate (GTN) [75]. This latter finding was properly interpreted with the hypothesis that GTN induces migraine, rather than by releasing CGRP, by a mechanism that operates downstream with respect to CGRP. The hypothesis implies that, if sumatriptan blocks headache and migraine attacks evoked by GTN [76–80], the drug should work by blocking not, or not solely, the release of CGRP by an inhibitory presynaptic action on sensory nerve terminals, but rather, or additionally, through some downstream and very basic mechanism, such as that activated by NO donors.

## Conclusion

Despite unprecedented advancement in migraine knowledge, essential issues remain unresolved in order to establish the vascular or non-vascular origin of migraine pain. Various findings challenge the vascular hypothesis. These, among others, include: (1) the still undetermined mechanism that, initiated by GTN and many other vasodilators, only after a significant time delay from their administration, provokes the migraine-like attack; (2) inconsistencies between the degree of vasodilatation and the severity of the headache [81, 82]; (3) the discrepancy between the potential of generating headaches between two similar vasodilatatory peptides [83, 84]; (4) the apparent lack of a simple correspondence between the subjective experience of throbbing pain and the arterial pulse [85]. In addition, current evidence cannot exclude that vasodilators used to trigger migraine attacks do not act via their vasoactive action, but rather through subtle changes in the brain and brainstem and that CGRP itself may act not, or not exclusively, at the vascular level, but at additional peripheral or central sites [86]. However, findings that emerge from studies in experimental animals, healthy volunteers, and migraine patients through the use of the provocation model of migraine underscore that ‘antidromic vasodilatation’, as defined more than a century ago by the imaginative vision of pioneers in physiological, neurobiological and pharmacological investigation, cannot as yet be discarded.
